# Arthroscopic Bioinductive Collagen Scaffold Augmentation in High-Risk Posterosuperior Rotator Cuff Tears: Clinical and Radiological Outcomes

**DOI:** 10.3390/jcm14248797

**Published:** 2025-12-12

**Authors:** Michael Kimmeyer, Geert Alexander Buijze, Madu Nayan Soares, Peter Rab, Antonio Gioele Colombini, Robin Diot, Arno Macken, Thibault Lafosse

**Affiliations:** 1 Alps Surgery Institute, Clinique Générale Annecy, 74000 Annecy, France; 2 Department of Orthopaedic and Trauma Surgery, University Medical Centre Mannheim, Medical Faculty Mannheim, University of Heidelberg, Theodor-Kutzer-Ufer 1-3, 68167 Mannheim, Germany; 3 Hand, Upper Limb, Peripheral Nerve, Brachial Plexus and Microsurgery Unit, Clinique Générale, 74000 Annecy, France; 4 Department of Orthopaedic Surgery, Amsterdam University Medical Center, University of Amsterdam, 1012 Amsterdam, The Netherlands; 5 Department of Human Movement Sciences, Vrije Universiteit Amsterdam, 1012 Amsterdam, The Netherlands; 6 Department of Orthopedic Sports Medicine, Klinikum Rechts der Isar, Technical University of Munich, 80333 Munich, Germany; 7 Department of Orthopedics and Trauma Surgery, University of Verona, 37129 Verona, Italy; 8 Department of Orthopedics and Traumatology, Lausanne University Hospital, 1005 Lausanne, Switzerland; 9 Department of Orthopedics and Sports Medicine, Erasmus Medical Center, 3015 Rotterdam, The Netherlands

**Keywords:** Regeneten patch, tendon augmentation, failure, Sugaya classification, magnetic resonance imaging

## Abstract

**Background/Objectives:** Bioinductive bovine collagen implants (BCI) have been introduced to enhance tendon biology and promote tissue regeneration in rotator cuff (RC) repairs. This study aimed to assess the clinical and radiological outcomes of arthroscopic posterosuperior rotator cuff (psRC) repair with BCI augmentation in full-thickness tears at increased risk of retear. **Methods:** This case series analyzed 30 patients with psRC tears who were classified as being at high risk of failure according to a predefined set of parameters, including patient history, radiological findings and intraoperative assessments, and the presence of psRC retears. All patients subsequently underwent arthroscopic psRC repair with BCI augmentation, compromising 21 primary and 9 secondary repairs. Clinical outcomes were assessed using Subjective Shoulder Value (SSV), American Shoulder and Elbow Surgeons (ASES) shoulder score, and Constant score at 6 and 12 months postoperatively. Tendon integrity was assessed using the Sugaya classification. **Results:** At 12 months, magnetic resonance imaging revealed complete tendon healing in 56.7%, partial healing in 16.7%, and insufficient healing in 26.7%. Significant improvements in SSV (45.3 to 83.5), ASES (40.6 to 77.8), and Constant score (36.6 to 71.7) were observed at 12 months postoperatively, with all outcome measures exceeding their respective minimally clinically important differences. Two patients (6.7%) developed secondary shoulder stiffness, and 1 patient (3.3%) required revision surgery for bicipital groove pain. **Conclusions:** Augmentation with a BCI in arthroscopic repair of high-risk psRC tears demonstrate promising short-term results. Patients achieve significant improvements in pain and shoulder function, accompanied by satisfactory tendon healing on MRI.

## 1. Introduction

Advances in surgical techniques and technological developments in the arthroscopic treatment of rotator cuff (RC) tears have expanded treatment options for patients of advanced age and those with compromised tendon integrity. But in cases of advanced RC degeneration, characterized by muscle fatty degeneration and atrophy, the risk of retear following primary surgery is significantly increased [1,2]. There might be surgical ways to support tendon healing [3,4,5]. Bioinductive bovine collagen implants (BCI) are suggested to create a favorable environment for a biological response of the host tissue that yields better healing of the RC [6], building on the positive histological findings from preclinical and human studies where BCIs facilitated the formation of mature, regularly oriented connective tissue indistinguishable from native tendon [5,6]. After BCIs were initially used primarily for partial supraspinatus (SSP) lesions [7,8], the range of indications expanded, and they are also used for patients with full-thickness posterosuperior rotator cuff (psRC) tears [9]. Several clinical studies have confirmed the effectiveness and clinical benefit of BCI augmentation in full-thickness psRC tears [9,10,11]. However, from a surgical, economic, and resource-conscious perspective, we believe that BCI augmentation is not indicated for every patient. Therefore, BCI augmentation in psRC repairs may be more appropriately reserved for patients considered at increased risk for retears.

The purpose of this study was to report on the use and indication criteria for BCI augmentation in psRC tears within a highly specialized shoulder arthroscopy clinic. This study aimed to assess the clinical and radiological outcomes of arthroscopic psRC repair with BCI augmentation in full-thickness tears at increased risk of retear. The hypothesis was that BCI augmentation is linked to improved clinical outcomes and promising tendon healing in high-risk psRC repairs.

## 2. Methods

### 2.1. Non-Comparative Single-Center Case Series

Data were prospectively collected in an institutional database and retrospectively analyzed. After approval from the institutional review board, all patients who underwent arthroscopic RC repair with BCI (Regeneten, Smith & Nephew, Andover, MA, USA) augmentation for psRC between May 2021 and June 2023 were included.

**Figure 1:**
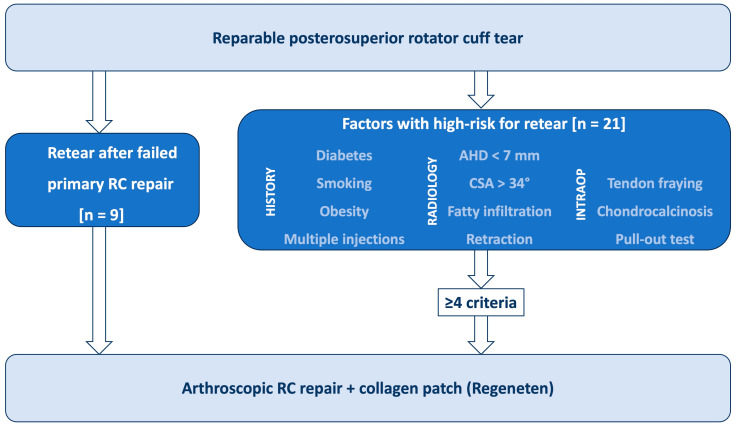
Diagram illustrating the therapy-determining risk factors for the use of a bioinductive bovine collagen scaffold in supraspinatus tendon repairs (RC: rotator cuff, AHD: acromiohumeral distance, CSA: critical shoulder angle, *n*: number).

BCI augmentation was performed in psRC tears which were considered at high risk of retear, including those with failed primary RC repair presenting with an SSP retear. Previous literature has identified multiple anamnestic, radiological, and intraoperative factors associated with increased failure risk after RC repair (Figure 1) [12,13]. Therefore, anamnestic factors such as age, diabetes mellitus, smoking, obesity, and corticoid injections were assessed [14]. Radiographic risk factors included a reduced acromiohumeral distance (AHD) < 7 mm [15], and an increased critical shoulder angle (CSA) >34 degrees [16]. Magnetic Resonance Imaging (MRI) and Computer Tomographic Arthrography (CTA) were used to evaluate psRC tendon retraction according to Patte [17], psRC fatty infiltration according to Goutallier [1], and psRC atrophy according to Thomazeau [2]. Patients with fatty infiltration >grade 2 and atrophy >grade 2 were excluded for psRC repair. Fatty infiltration grade 2 or less and muscle atrophy grade 2 or less of the psRC muscle were considered risk factors [1,2,18]. Intraoperative risk factors included poor tendon quality such as tendon fraying, tendon calcifications (chondrocalcinosis), limited tendon mobility [18], and a positive pull-out test, in which the tendon failed to withstand axial pull on the suture [19].

In summary, the BCI was used in patients with a history of failed SSP repair or in those presenting with at least four of the defined risk factors as shown in Figure 1. Patients with concurrent subscapularis tendon (SSC) tears were not excluded from the study. All surgeries were performed by two experienced shoulder surgeons (T.L., G.A.B.).

### 2.2. Surgical Technique and Rehabilitation Protocol

The operations were performed under general anesthesia and an interscalene block, with the patient positioned in the beach chair position. Arthroscopic RC repair was carried out using either a single-row or double-row technique, depending on the size of the rupture. After fixation of the RC, a BCI was placed on the repaired SSP tendon. An assistant was required to hold the collagen scaffold in the correct position during placement. The specialized cannulas included in the kit set facilitated the insertion of the stapling device. The staples were carefully positioned to avoid placing them too close to the edges of the scaffold to prevent rupture. An additional portal was often necessary for the placement of bone anchors. Further pathologies were treated arthroscopically. The psRC repair was performed using either a single-row or double-row technique as necessary. Tenotomy or tenodesis of the long head of the biceps tendon (LHBT) and subacromial decompression were performed in all patients. Resection of the lateral clavicle was conducted in patients with osteoarthritis of the acromioclavicular (AC) joint.

Postoperatively, patients were immobilized in a 30° abduction shoulder sling for 6 weeks. Pendulum exercises and passive, pain-free mobilization was permitted. Active rehabilitation commenced at 6 weeks postoperatively, with resistance exercises introduced after 3 months. Return to sport was determined individually in collaboration with the physiotherapist, trainer, and patient.

### 2.3. Outcome Variables

Preoperatively and at 6 (FU6) and 12 (FU12) months postoperatively, Patient-Reported Outcome Measures (PROMs) including Visual Analog Scale (VAS) for pain-score, Subjective Shoulder Score (SSV), Disabilities of the Arm, Shoulder and Hand (DASH) score (DASH) [20], and American Shoulder and Elbow Surgeons (ASES) shoulder score [21] were assessed. The minimally clinically important difference (MCID) was calculated for each outcome variable [22]. The active Range of Motion (ROM) of both shoulders (abduction, anterior elevation, external rotation, internal rotation), SSP tests during clinical examination (full can and empty can; references), and the Constant score [23], were assessed during the follow-up examination. For the full can and empty can tests, pain was assessed according to the Constant score (15 points for no pain, 10 points for mild pain, 5 points for moderate pain, and 0 points for severe pain), and force was assessed using the 5-point Medical Research Council (MRC) score for muscle strength.

**Figure 2:**
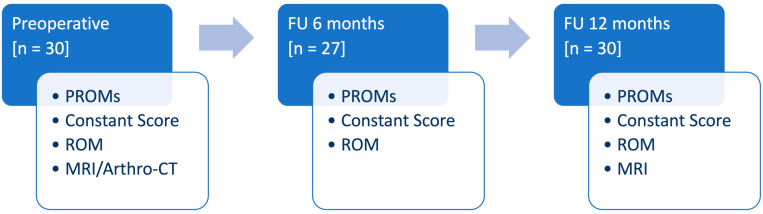
Diagram illustrating the study design, including the clinical preoperative and follow-up evaluation (PROMs: patient-reported outcome measures, ROM: range of motion, MRI: magnetic resonance imaging, CT: computer tomographic imaging, FU: follow-up, *n*: number).

At FU12, MRI was performed and tendon healing of the SSP was analyzed in T2 weighted images according to the Sugaya classification [24]. The analysis was performed by two surgeons (M.K., P.R.). In cases of discrepancy, consensus was reached in consultation with the senior author (T.L.). Complications were considered for adverse events related to the surgery. Revision surgery was defined as any unplanned additional surgery to the same shoulder related to the initial intervention. The study design, including the clinical preoperative and follow-up evaluations, is presented in Figure 2.

### 2.4. Statistical Evaluation

Statistical analysis was performed using R version 4.3.1 (R foundation for statistical computing, Vienna, Austria) and RStudio software (Version 4.4.0, Rstudio, Boston, MA, USA). Nominal variables were presented by numbers and proportions. Metrical variables were tested for normality using the D’Agostino-Pearson omnibus test and presented using mean and Standard Deviation (SD). To compare pre- and postoperative variables, the paired-samples *t*-test for normally distributed metric data and the Wilcoxon Signed-Rank test for non-normally distributed matric or ordinal data was applied. Point-Biserial Correlation was used to correlate normally distributed metric preoperative and perioperative factors with retears at FU12. Spearman’s Rank Correlation was used to analyze the relation between ordinal or non-normally distributed metric factors and retears at FU12. Lastly, Fisher’s Exact Testing was used to analyze the relation between binary factors and retears at FU12. The significance level was set at *p* = 0.05.

## 3. Results

### 3.1. Demographic Parameters and Intraoperative Characteristics

A total of 30 patients were included, of whom 21 (70%) underwent primary psRC repair and 9 (30%) underwent revision repair after psRC retear. All patients completed the 12-month follow-up. The mean age was 59.5 ± 8.9 years, with 17 males (56.7%) and 13 females (43.3%). 10 patients (33.3%) were categorized into each group of the Patte classification. A double-row refixation technique was performed in 26 patients (86.7%), while a single-row refixation was performed in 2 patients with small sized tendon tears (6.7%), and side-to-side RC repair in 2 patients with longitudinal intratendinous tears (6.7%). SSC tears requiring refixation were observed in 13 patients (43.3%). Of these, double-row refixation was performed in 6 patients (46.2%), and single-row refixation in 7 patients (53.8%). Detailed demographic and intraoperative characteristics are summarized in Table 1 and Table 2.

### 3.2. Follow-Up Evaluation

**Figure 3:**
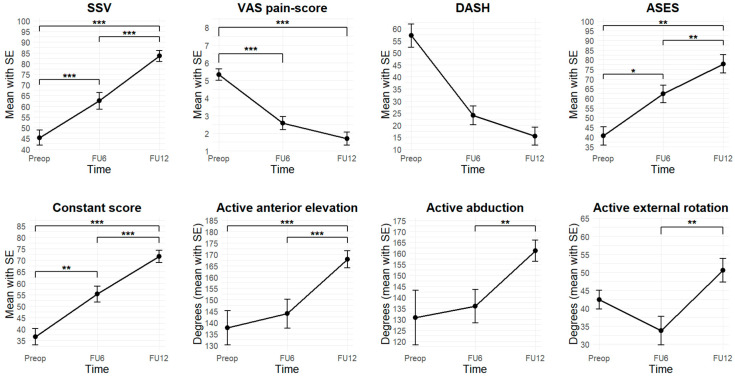
Evolution of clinical outcome parameters (Preop: preoperatively, FU6: follow-up 6 months postoperatively, FU12: follow-up 12 months postoperatively, SSV: Subjective Shoulder Value, VAS: Visual Analog Scale, DASH: Disabilities of Arm, Shoulder and Hand, ASES: American Shoulder and Elbow Surgeons, *: *p* < 0.05, **: *p* < 0.01, ***: *p* < 0.001).

After 6 months, 27 patients (90.0%) were available for follow-up examination. At FU12, 30 of the patients (100%) were followed up. MRI was performed in 29 patients (96.7%). In 1 patient (3.3%), postoperative MRI was not possible due to a cardiac pacemaker, however an Arthro-CT scan was carried out. Figure 3 illustrates the evolution of clinical outcome parameters from preoperative, to 6 months postoperative, to 12 months postoperative.

### 3.3. Clinical Scores

All clinical scores improved at FU6 and continued to show significant further improvement at FU12. All measures exceeded MCID thresholds. Detailed trajectories are shown in Figure 3.

At FU6, there was an improvement in SSV from 45.3 ± 18.9 to 62.5 ± 19.9 (*t* = 4.4, *p* < 0.001). At FU12, there was an improvement to 83.5 ± 14.2 compared to both preoperatively (*t* = 510.0, *p* < 0.001) and FU6 (*t* = 7.0, *p* < 0.001). At FU6, ASES improved from 40.6 ± 19.0 to 62.3 ± 23.0 (*V* = 17.0, *p* = 0.02). At FU12, an improvement to 77.8 ± 24.6 was observed compared to preoperatively (*V* = 7.0, *p* = 0.002) and compared to FU6 (*t* = 3.2, *p* = 0.004). At FU6, the Constant score improved from 36.6 ± 16.9 to 55.2 ± 18.2 (*t* = 3.6, *p* = 0.002). At FU12, an improvement to 71.7 ± 14.8 was observed compared to both preoperatively (*V* = 0.0, *p* < 0.001) and FU6 (*V* = 24.0, *p* < 0.001). At FU6, the DASH score improved from 57.8 ± 11.9 to 24.0 ± 16.7 (*V* = 6.0, *p* = 0.25). At FU12, there was a significant improvement to 15.4 ± 18.4 compared to preoperatively (*V* = 15.0, *p* = 0.06). At FU6, the VAS pain-score improved from 5.3 ± 1.8 to 2.6 ± 1.9 (*t* = 6.1, *p* < 0.001). At FU12, an improvement to 1.7 ± 2.0 was observed, compared to preoperative values (*V* = 426.5, *p* < 0.001), but not to FU6 (*V* = 138.5, *p* = 0.08).

### 3.4. ROM

Active ROM showed no significant improvement at FU6 compared to preoperatively but demonstrated significant improvement at FU12. At FU6, no improvements were observed in active anterior elevation, abduction, external rotation, or internal rotation. However, at FU12, all parameters showed significant improvement. Active anterior elevation increased to 167.8 ± 20.4 degrees compared to both preoperatively (*V* = 34.0, *p* < 0.001) and FU6 (*V* = 4.5, *p* < 0.001).

Active anterior elevation improved at FU12 (167.8 ± 20.4 degrees) compared to both preoperative (*V* = 34.0, *p* < 0.001) and FU6 (*V* = 4.5, *p* < 0.001) values. Abduction improved significantly at FU12 (161.2 ± 26.1 degrees) compared to FU6 (*V* = 17.0, *p* = 0.002), and active external rotation significantly increased at FU12 (50.5 ± 17.7 degrees) compared to FU6 (*t* = 5.2, *p* < 0.001). Similarly, active internal rotation improved to a median of T10 (range: sacrum–T1), showing significant improvement over both preoperative values (*V* = 40.0, *p* = 0.001) and FU6 (*V* = 23.0, *p* = 0.004). Detailed results are illustrated in Figure 3.

### 3.5. Tendon Healing

**Figure 4:**
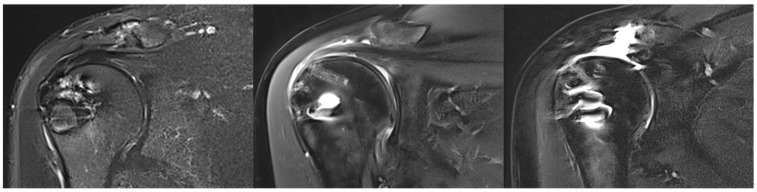
Illustration of supraspinatus tendon healing according to Sugaya. (**Left**): complete healing (Sugaya 1), (**middle**): unclear healing (Sugaya 3), (**right**): non-healing (Sugaya 5).

At FU12, MRI confirmed satisfactory and complete tendon healing in 17 patients (56.7%) classified as Sugaya type 1 (11 patients, 36.7%) and Sugaya type 2 (6 patients, 20.0%). Partial tendon healing, classified as Sugaya type 3, was observed in 5 patients (16.7%). Insufficient tendon healing was found in 8 patients (26.7%) classified as Sugaya type 4 (2 patients, 6.7%) and Sugaya type 5 (6 patients, 20.0%). The rate of insufficient healing was 44.4% in the secondary repaired group and 19.0% in the primary repaired group (*p* = 0.20). There was no correlation between preoperative or intraoperative risk factors and retear at FU12. Moreover, there were no significant differences in clinical outcomes between healed and non-healed SSP tendons, with both cohorts demonstrating significant improvement. Figure 4 provides example MRI images corresponding to Sugaya types 1, 3, and 5.

### 3.6. Complications and Revision Surgery

In this cohort, two patients (6.7%) had secondary shoulder stiffness at both FU6 and FU12. Both patients were female with isolated Patte type 1 SSP tears. Slight improvements in clinical scores and ROM were observed in both patients when comparing FU12 to FU6.

No revision surgeries were related to the RC repair and BCI augmentation. In one patient (3.3%), surgery was performed 9 months postoperatively due to chronic pain in the bicipital groove after self-locking (y-shape) tenodesis of the LHBT, which was managed with a subpectoral tenodesis of the LHBT.

## 4. Discussion

This case series study reports on BCI augmentation for chronic, full-thickness psRC tears in high-risk patients at a specialized shoulder clinic. Thirty patients (mean age: 59.5) were selected using defined pre- and intraoperative criteria. Clinical scores (SSV, VAS, and ASES) improved significantly at 6 and 12 months. Imaging showed complete healing in 56.7% and insufficient healing in 26.7%. Despite the high-risk cohort, outcomes were clinically favorable with a moderate retear rate. In this cohort, both healed and non-healed SSP tendons showed significant clinical improvement, with no notable difference in outcomes. In contrast to previous studies reporting better outcomes in Sugaya types 1 and 2 [25], our findings suggest comparable clinical improvement across all Sugaya types. In addition to tendon healing, postoperative stiffness is an important consideration when evaluating BCI augmentation. In the study by Yeazell et al., the use of a BCI was associated with a secondary shoulder stiffness rate of 13% [26]. In contrast, both our study and that of McIntyre et al. reported notably lower rates of shoulder stiffness, 7% and 1%, respectively [10]. These differences should be interpreted with caution, as Yeazell et al. investigated partial-thickness tears, which are not directly comparable to full-thickness tears. Additionally, their shorter follow-up period of up to 6 months may have contributed to the higher reported stiffness rate.

Numerous studies have examined the use of BCI augmentation in full-thickness psRC tears, and consistently report favorable clinical outcomes with high healing rates up to 96% [9,10,11,27,28]. A recent systematic review by Warren et al. reported a retear rate of 8.3% following full-thickness RC repair augmented with BCI [28]. Thon et al. reported high healing rates (96%) and good functional outcomes after 24 months in their case series study of 23 patients [27]. Bokor et al. reported excellent clinical and radiological outcomes, with all repairs intact and normal footprint restoration, likely due to less compromised tendon quality [9]. McIntyre et al. also observed significant clinical improvements and a low retear rate (5%) in their prospective, multicenter registry study [10]. Camacho-Chacon et al. evaluated the outcomes in patients with RC tears treated with BCI augmentation and showed significant improvements in clinical scores and complete tendon healing in 27 patients (90%) [29]. Histological examination of biopsies at 6 months postoperatively confirmed that the BCI effectively promotes tendon regeneration and integrates well with surrounding tissue, showing consistent results across different tear types and sizes [29]. In a recently published randomized controlled trial, Ruiz Iban et al. demonstrated a significantly lower retear rate (8%) in patients receiving BCI augmentation compared with standard repair, indicating that the implant can effectively enhance tendon healing in medium-to-large tears [11]. While the clinical outcomes reported in these studies are comparable to ours, the retear rates are notably lower. The discrepancy likely reflects differences in patient selection, as our cohort consisted of individuals with poorer tendon quality and a higher biological risk of retear. For example, Ruiz Iban et al. included only isolated SSP tears and excluded patients with SSC tears or revision surgery [11], whereas our study intentionally incorporated high-risk patients, including those with failed prior repairs. In our revision subgroup, good tendon healing reached 56%, which, although reduced, still indicates potential benefit.

Cost considerations also play a role when expanding the indication to high-risk patients. While cost-effectiveness analyses suggest that higher upfront cost of the implant may be offset by reduced rehabilitation time, fewer revision surgeries, and improved productivity [30,31], long-term data are still needed to determine whether BCI augmentation is truly cost-effective in routine practice. In addition, cost-effectiveness analyses must be interpreted within the context of varying national healthcare systems and reimbursement structures. Until stronger evidence is available, a selective use of BCI, particularly in revision cases and in carefully chosen primary cases at high risk of retear, appears most appropriate, as these groups are most likely to benefit from the biological effects of the implant.

Importantly, this is the first study to specifically investigate BCI augmentation in a high-risk population undergoing RC repair, including those with recurrent tears following failed prior repairs. This represents a novel and clinically relevant contribution, as such patients are underrepresented in the existing literature. Although our findings show promising clinical outcomes and healing rates, further validation is required, and direct comparison with previous studies is limited by our distinct inclusion criteria.

This study has several limitations. The relatively small sample size of 30 patients and the short follow-up period may limit the generalizability of the findings and cannot capture long-term outcomes or late complications. This study population was highly specific, consisting of selected high-risk patients and including both primary and revision cases, which further restricts the external applicability of the results. The definition of “high-risk” was based on a pragmatic threshold of ≥4 risk factors, as no validated scoring system currently exists for stratifying rotator cuff healing risk in the context of BCI augmentation. This threshold was therefore chosen subjectively by combining multiple anamnestic, radiological, and intraoperative parameters known to be individually associated with impaired tendon healing. Moreover, these risk factors were analyzed individually without accounting for potential interactions, limiting the identification of independent predictors of retear. Several concomitant procedures such as SSC repair, LHBT tenotomy or tenodesis, subacromial decompression, and distal clavicle resection were performed based on surgeon preference. Although none of these co-interventions showed a significant effect on clinical or radiological outcomes in this cohort, this observation is limited by the small sample size and should be interpreted with caution. Larger prospective studies with longer follow-up and multivariate analysis are needed to confirm these findings and better define optimal patient selection.

## 5. Conclusions

Augmentation with a BCI in arthroscopic repair of high-risk psRC tears demonstrate promising short-term results. Patients achieve significant improvements in pain and shoulder function, accompanied by satisfactory tendon healing on MRI.

## Figures and Tables

**Table 1:** Patient characteristics (*n*: number, SD: standard deviation, BMI: body mass index, CSA: critical shoulder angle, AHD: acromiohumeral distance, mm: millimeter, SSP: supraspinatus muscle).

Patient Characteristics	
Patients, *n* (%)	30 (100)
Gender	
Male, *n* (%)	17 (56.7)
Female, *n* (%)	13 (43.3)
Affected dominant side, *n* (%)	18 (60.0)
Age, mean years (SD)	59.5 (8.9)
BMI, mean kg/m^2^ (SD)	26.1 (5.0)
Overweight, *n* (%)	17 (56.7)
Obesity, *n* (%)	7 (23.3)
Diabetes, *n* (%)	2 (6.7)
Smoking, *n* (%)	7 (23.3)
Preoperative injections, *n* (%)	30 (100.0)
Failed SSP repair, *n* (%)	9 (30.0)
CSA > 34°, *n* (%)	18 (60.0)
AHD < 7 mm, *n* (%)	13 (43.3)
SSP fatty infiltration	
Goutallier 0, *n* (%)	24 (80.0)
Goutallier 1, *n* (%)	5 (16.7)
Goutallier 2, *n* (%)	1 (3.3)
SSP atrophy	
Thomazeau 1, *n* (%)	28 (93.3)
Thomazeau 2, *n* (%)	2 (6.7)
SSP calcifications, *n* (%)	10 (33.3)

**Table 2:** Intraoperative classification of the rotator cuff tears (*n*: number, SD: standard deviation, SSP: supraspinatus muscle, ISP: infraspinatus muscle, SSC: subscapularis muscle).

Intraoperative Classification of the Rotator Cuff Tear	
SSP tear classification	
Patte 1, *n* (%)	10 (33.3)
Patte 2, *n* (%)	10 (33.3)
Patte 3, *n* (%)	10 (33.3)
ISP tear classification	
No tear	8 (26.7)
Patte 1, *n* (%)	15 (50.0)
Patte 2, *n* (%)	5 (16.7)
Patte 3, *n* (%)	2 (6.7)
SSC tear classification	
No tear	17 (56.7)
Lafosse 1, *n* (%)	3 (10.0)
Lafosse 2, *n* (%)	5 (16.7)
Lafosse 3, *n* (%)	2 (6.7)
Lafosse 4, *n* (%)	3 (10.0)

## Data Availability

Patient consent does not include publication of the data, so publication of the raw data is not possible. The datasets generated and/or analyzed during the current study are not publicly available due to not having written consent of the patients but are available from the corresponding author on reasonable request.

## Abbreviations

AC	Acromioclavicular (joint)
ASES	American Shoulder and Elbow Surgeons (score)
AHD	Acromiohumeral Distance
BCI	Bioinductive Collagen Implant
BMI	Body Mass Index
CSA	Critical Shoulder Angle
CT	Computed Tomography
DASH	Disabilities of the Arm, Shoulder and Hand (score)
FU6	Follow-Up at 6 Months
FU12	Follow-Up at 12 Months
LHBT	Long Head of the Biceps Tendon
MCID	Minimally Clinically Important Difference
MRI	Magnetic Resonance Imaging
MRC	Medical Research Council (muscle strength score)
PROMs	Patient-Reported Outcome Measures
psRC	posterosuperior Rotator Cuff
RC	Rotator Cuff
ROM	Range of Motion
SD	Standard Deviation
SSC	Subscapularis (muscle/tendon)
SSP	Supraspinatus (muscle/tendon)
SSV	Subjective Shoulder Value
VAS	Visual Analog Scale (for pain)
